# Supplementary material for: The Enzyme Gene Expression of Protein Utilization and Metabolism by Lactobacillus helveticus CICC 22171
Source: Microorganisms. 2022 Aug 26;10(9):1724. doi: 10.3390/microorganisms10091724 (PMC9501481; doi:10.3390/microorganisms10091724)
Supplement: Supplementary file 1 [file microorganisms-10-01724-s001.zip › microorganisms-1863331-supplementary.pdf]

Supplementary material

Table S1. Primer sequence related to PCR

Number	Sequence(5'-3')	GC%	TM
1-F	ATGAAGAGTTATATGAAAAAAGTTGC	26.92	51.71
1-R	TTACTTAGTAGCACCAGTTGAAACGT	38.46	56.44
2-F	AAATTGTTGCACATTTACTGAAGA	29.17	51.03
2-R	TTTGTATTGCCAGTTAGTGTTGAT	33.33	52.73
3-F	TTAAACGTACTTCTTGCCTG	40.00	51.30
3-R	TTGGTAACGTTTGATAAGGTAAGC	37.50	54.44
4-F	TTAATTTTCACGTTGTCTTGCGTC	37.50	54.44
4-R	ATGGCTGAAAAGAAAGAAACTGCT	37.50	54.44
5-F	CTATTGAATTCTAATACGTG	30.00	47.20
5-R	ATGTGGAAAACAATCTTACG	35.00	49.25
6-F	TTAATCACTCTCCTTTTC	33.33	45.79
6-R	ATGACAAAAGAAATTATCCAAA	22.73	46.49
7-F	TTACTCCCCGCTTGCTGCTG	60.00	59.50
7-R	TTGGAAAAGCAGAGTGATCTATTACTAG	35.71	56.69
8-F	TTAATGAATTCCTAACTCAATC	27.27	48.35
8-R	TTGGGTAAACGAGATTCAAATA	31.82	50.22
9-F	ATGGCGATTCCAACAAGAAG	45.00	53.35
9-R	TTACTTACCAATCAAACTCTCGAA	33.33	52.73
10-F	TTACCAAATGACTACGCGCTTAGCAG	46.15	59.59
10-R	ATGAATTTAGCAAAAATCCG	30.00	47.20
11-F	TTAAGCAAGTGAATCCCAT	36.84	48.69
11-R	ATGGCTCATGAATTAACTG	36.84	48.69
12-F	TTAATACTTAAATGCTAAAGCACCC	32.00	53.02
12-R	ATGGCAAAAGAAATTAATAACGAT	25.00	49.32
13-F	ATGCAAAAAGAACAAGAAATTCAAAT	23.08	50.13
13-R	TTAAAACTTTTTAAAGCTATCTAA	16.67	45.90
14-F	TTGATTACAATTAAATCAATTA	13.64	42.76
14-R	CTAGTCATCCATCTCATC	44.44	50.34
15-F	ATGAAATATAACCAATATGCT	23.81	45.85
15-R	TTATTTTTCATAAAACTTGA	15.00	41.05
16-F	ATGAAAAGAAGAACAACA	27.78	43.51
16-R	TTATTCACTTAAGTACTTAG	25.00	45.15
17-F	TTAGGCTTCTTTTGTTAAT	26.32	44.37
17-R	ATGAATTTAGACTACAAGA	26.32	44.37
18-F	TTAGTCGAGCAAATCGTACTTC	40.91	53.95
18-R	ATGAAACAAACAGAATGTACTACTAT	26.92	51.71
19-F	ATGAGTTTTTACGATTTAAGTTACGA	26.92	51.71
19-R	TTACCAATATTTCTCAAGTGGGCGTT	38.46	56.44
20-F	CTAAAATTTTCTCACCCCATGC	40.91	53.95
20-R	ATGGCGGTAGCAGAAGCTCA	55.00	57.45
21-F	TTATCTGCCTCCGAAAAGCATCCG	50.00	59.57

21-R	ATGTTTTTTAGTAAAAAGATAAAACGAGCT	23.33	52.80
22-F	TTATTCACCTTTAAAAGTGC	30.00	47.20
22-R	ATGAAAACTGGTACTAAAATCA	27.27	48.35
23-F	GATGCGCCATCAATCTTCTT	45.00	53.35
23-R	GGTACTTCAATGGCTTCTCC	50.00	55.40

Table S2. Primer sequences related to Real-time PCR

Name	Sequence(5'-3')	GC%	TM
16S rRNA-F	CTTCTTCACCAACAACAG	44.44	50.34
16S rRNA-R	CTTACCAAGGCAATGATG	44.44	50.34
PRTP-1-F	TTAAAAGGTCCCGACGAC	50.00	52.62
PRTP-1-R	CGCAACTCCCACCACATA	55.56	54.9
PRTP-2-F	ACGCCTTCGTTTATGATGC	47.37	53.01
PRTP-2-R	AGTAATTTGACCCTTGTGCC	45.00	53.35
PRTM-F	TGCTGCCTGCTCAAATAG	50.00	52.62
PRTM-R	TGTTCCAAAGCACGGTTA	44.44	50.34
PRTH-1F	TAACGCTAACGCTACTATC	42.11	50.85
PRTH-1R	TGAGTTCTACACCAGGAT	44.44	50.34
PRTH-2F	ACAATGAACGACAAGAATGG	40.00	51.3
PRTH-2R	ATGGCTAATATCAGGTTGAATAC	34.78	52.42
OPPA-1-F	TGCCAAGATGAACCACAA	44.44	50.34
OPPA-1-R	AGCTGCAAGACGGATAGT	50.00	52.62
OPPA-2-F	TGGAAGGATGGCAGAAAT	44.44	50.34
OPPA-2-R	ATGGTTTGCCGTTAGGTT	44.44	50.34
OPPB-F	CTAACACCGACCCTAAACA	47.37	53.01
OPPB-R	CAAGAGGAATCGCAATACTA	40.00	51.3
OPPC-F	AGGCTACTCTTCCACCTTCTG	52.38	57.57
OPPC-R	ATTTCGGTAACTTGTGCTCTG	42.86	53.66
OPPD-F	AGTTACAGGGGACATTCTTT	40.00	51.3
OPPD-R	TTTCTTCAGGTCTAGGCATT	40.00	51.3
OPPF-F	GTCACTAAGGCAAGCAATCG	50.00	55.4
OPPF-R	ATTCTTTGACGCTGACCACC	50.00	55.4
DTPT-F	TCACTGAAATGTGGGAGC	50.00	52.62
DTPT-R	ACCGCCGTAGAAGACT	56.25	51.71
PEP N1-F	ACTTCGCTTTGGACATTG	44.44	50.34
PEP N1-R	GCCATAAGTTGTCCCACC	55.56	54.9
PEP N2-F	TAAGGCTACAAGAAATGCG	42.11	50.85
PEP N2-R	TGGGAAACAGAATGACAGG	47.37	53.01
